# Machine Learning for Prediction of Outcomes in Cardiogenic Shock

**DOI:** 10.3389/fcvm.2022.849688

**Published:** 2022-05-06

**Authors:** Fangning Rong, Huaqiang Xiang, Lu Qian, Yangjing Xue, Kangting Ji, Ripen Yin

**Affiliations:** Department of Cardiology, The Second Affiliated Hospital and Yuying Children's Hospital, Wenzhou Medical University, Wenzhou, China

**Keywords:** cardiogenic shock, intensive care unit, machine learning, CoxBoost, predictive model

## Abstract

**Objective:**

The management of cardiogenic shock (CS) in the elderly remains a major clinical challenge. Existing clinical prediction models have not performed well in assessing the prognosis of elderly patients with CS. This study aims to build a predictive model, which could better predict the 30-day mortality of elderly patients with CS.

**Methods:**

We extracted data from the Medical Information Mart for Intensive Care III version 1.4 (MIMIC-III) as the training set and the data of validation sets were collected from the Second Affiliated Hospital and Yuying Children's Hospital of Wenzhou Medical University. Three models, including the cox regression model, the Least Absolute Shrinkage and Selection Operator (LASSO) regression model, and the CoxBoost model, were established using the training set. Through the comparison of area under the receiver operating characteristic (ROC) curve (AUC), C index, net reclassification improvement (NRI), integrated discrimination improvement (IDI), and median improvement in risk score, the best model was selected. Then for external validation, compared the best model with the simplified acute physiology score II (SAPSII) and the CardShock risk score.

**Results:**

A total of 919 patients were included in the study, of which 804 patients were in the training set and 115 patients were in the verification set. Using the training set, we built three models: the cox regression model including 6 predictors, the LASSO regression model including 4 predictors, and the CoxBoost model including 16 predictors. Among them, the CoxBoost model had good discrimination [AUC: 0.730; C index: 0.6958 (0.6657, 0.7259)]. Compared with the CoxBoost model, the NRI, IDI, and median improvement in risk score of other models were all<0. In the validation set, the CoxBoost model was also well-discriminated [AUC: 0.770; C index: 0.7713 (0.6751, 0.8675)]. Compared with the CoxBoost model, the NRI, IDI, and median improvement in risk score of SAPS II and the CardShock risk score were all < 0. And we constructed a dynamic nomogram to visually display the model.

**Conclusion:**

In conclusion, this study showed that in predicting the 30-day mortality of elderly CS patients, the CoxBoost model was superior to the Cox regression model, LASSO regression model, SAPS II, and the CardShock risk score.

## Background

Cardiogenic shock (CS) is an extremely serious clinical condition and occurs as a consequence of cardiac pump failure, which has a high mortality (1, 2). In recent decades, the prevalence of CS has increased from 4.1 to 7.7%, and the mortality rate is up to 40% (3–5). Age itself increases the risk of CS associated with myocardial infarction, and advanced age itself is an independent risk factor for CS (6). This suggested that for the elderly, the risk of CS will be higher. The WHO defines the elderly as over 65 years old; as this aging population continues to grow, it is crucial to pay attention to issues related to this group. Therefore, the management of CS in the elderly is a huge clinical challenge (7, 8). Finding an efficient method to early evaluate the prognosis is helpful for clinicians to identify high-risk patients in time, and make better medical decisions in clinical practice.

However, in the elderly population, information on the prevalence, determinants, and prognostic factors of CS is scarce. Some researchers have constructed risk prediction models to facilitate risk assessment and predict the mortality of CS, such as the CardShock risk score (9, 10), but these models still have some shortcomings, such as the calculation methods are complicated, or the included predictors of some models are not all objective data. For example, in the CardShock risk score, the evaluation of the patients' consciousness depends on the subjective judgment of clinicians. Besides, they are not completely built for the elderly population, the evaluation effect of the prognosis of the elderly needs further assessment.

Compared with traditional forecasting methods, novel machine learning techniques can process high-dimensional data, identify complex relationships between variables and develop precise clinical prediction models, which have received more and more attention and recognition from clinicians (11, 12). There are many studies that apply machine learning to the evaluation of a variety of diseases and with good results (13–15). It has also been used in other medical auxiliary disciplines, such as imaging and anesthesiology (16, 17). Hou et al. (18) used XGboost machine learning to predict 30-days mortality of patients with sepsis. Weyer and Binder (19) developed a weighting approach for judging the effect of patient strata on high-dimensional risk prediction signatures using CoxBoost. Therefore, we hope to use the CoxBoost method to help us better predict the prognosis of elderly patients with CS.

Based on the Medical Information Mart for Intensive Care III (MIMIC-III) database and the Second Affiliated Hospital and Yuying Children's Hospital of Wenzhou Medical University (WMU), the goal of this study is to build a predictive model using CoxBoost machine learning, which can predict the 30-day mortality of elderly patients with CS.

## Methods

### Data Source and Research Population

We extracted data from MIMIC-III database version 1.4 as the training set, which is a public and free intensive care unit (ICU) database (20). Since the MIMIC-III database was approved by the Institutional Review Boards (IRB) of Beth Israel Deaconess Medical Center (Boston, MA) and the Massachusetts Institute of Technology (Cambridge, MA), IRB approval from our institution was exempted. Data of validation sets were collected from the patient data of ICU at the Second Affiliated Hospital and Yuying Children's Hospital of WMU. The Medical Ethics Committee of the Second Affiliated Hospital and Yuying Children's Hospital, WMU approved the use of the patient data of the Second Affiliated Hospital and Yuying Children's Hospital (Ethical Review Number: 2021-K-71-01).

**Figure 1:**
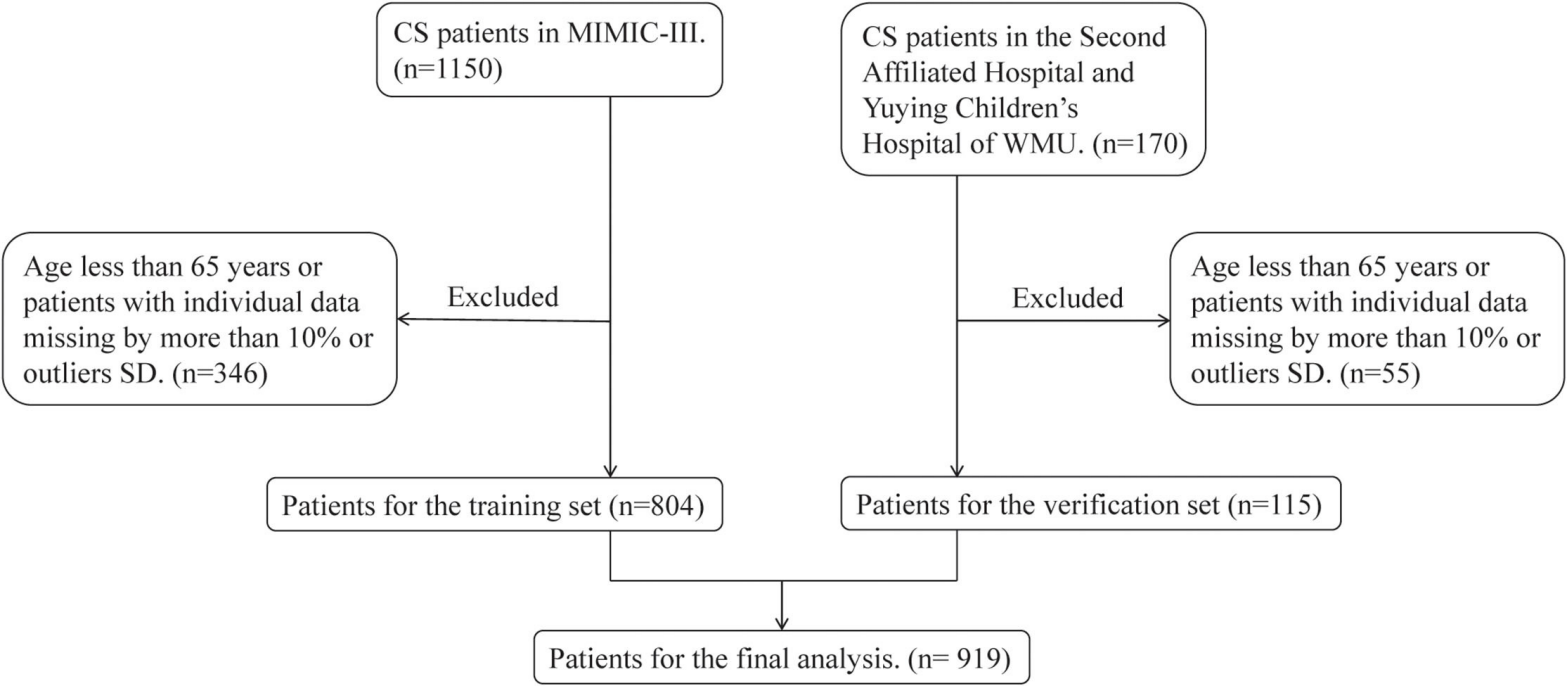
Flowchart of patient selection.

The inclusion criteria were as follows: (1) ICU admissions age ≥ 65 years; (2) CS patients in MIMIC-III database and the Second Affiliated Hospital and Yuying Children's Hospital of WMU. Among them, patients from the MIMIC-III database were diagnosed according to the International Classification of Diseases, Ninth Revision (ICD-9) diagnostic code 785.51 or 998.01, and patients from the Second Affiliated Hospital and Yuying Children's Hospital of WMU were selected by unified diagnostic criteria: minimum systolic blood pressure (SBP) <90 mmHg, or need of vasopressors therapy or signs of hypoperfusion. Only records of the first ICU visit were selected for analysis to eliminate duplicate data. The specific process was shown in Figure 1.

### Data Extraction and Outcome

We extracted the following information of patients on the first day of admission: (1) demographic information such as age, gender, ethnicity, and pathogeny; (2) vital sign data including heart rate, SBP, mean blood pressure (MBP), diastolic blood pressure (DBP), respiratory rate, temperature, and arterial oxygen saturation (SpO_2_); (3) laboratory data including white blood cell (WBC) count, red cell distribution width (RDW), hemoglobin, hematocrit, platelet, activated partial thromboplastin time (APTT), international normalized ratio (INR), prothrombin time (PT), anion gap, bicarbonate, glucose, blood lactic acid, serum creatinine, serum urea nitrogen, serum sodium, and serum potassium; (4) comorbidities including congestive heart failure, atrial fibrillation, coronary heart disease, renal failure, liver disease, stroke, tumor, chronic obstructive pulmonary disease (COPD), acute respiratory distress syndrome (ARDS), and pneumonia; (5) traditional severity scores including simplified acute physiology score II (SAPSII); (6) As well as ejection fraction and the use of the vasopressor. If multiple measurements were made in the first 24 h, the vital signs and the laboratory data were based on the results of the first examination. The endpoint was 30-day mortality.

### Missing Data

Variables with missing values ≥40% were excluded directly. We used multiple interpolations to deal with missing variables <40%. Patients with individual data missing by more than 10% or outliers [values above the mean of 3 standard deviations (SD)] were excluded. In the training set and the verification set, we carried out the above data processing respectively.

### Statistical Analysis

The continuous variables were expressed as mean ± SD of the normal distribution, while the non-normal continuous variables were expressed as median (interquartile range). Wilcoxon W test or Kruskal Wallis test was used to assess the differences among groups. The variance inflation factor (VIF) was calculated to verify whether multicollinearity existed.

In the model development stage, three models were established using the training set: the cox regression model, the Least Absolute Shrinkage and Selection Operator (LASSO) regression model, and the CoxBoost model. In the Cox regression model, we used the monofactor analysis to pick out targeted predictors and screened the predictors twice using multiple regression analysis. After two rounds of screening, the predictive factors were finally incorporated into the Cox regression model. The main idea of LASSO is to construct a first-order penalty function to obtain a refined model and to carry out feature screening by finally determining the coefficients of some variables to be 0. In the LASSO regression model, we determined the cutoff value based on ten-folds cross-validation to select the most useful predictive variables to form the LASSO regression model. CoxBoost (https://github.com/binderh/CoxBoost) is used to fit a Cox proportional hazards model by componentizing likelihood-based boosting, which is particularly suitable for models with a large number of predictors and allows for mandatory covariates with unpenalized parameter estimates. In contrast to gradient boosting, CoxBoost is not based on gradients of loss functions but adapts the offset-based boosting approach for estimating Cox proportional hazards models (21). According to the CoxBoost machine learning analysis of the importance of the predictors, we select targeted variables incorporated into the CoxBoost regression model.

These three models were evaluated together with the classic SAPSII score model. The area under receiver operating characteristic (ROC) curve (AUC), C index, net reclassification improvement (NRI), integrated discrimination improvement (IDI), and median improvement in risk score were used to choose the best model. In the validation set, AUC, C index, NRI, IDI, and median improvement in risk score were also used to evaluate the CoxBoost model, SAPSII score model, and the CardShock risk score model for external validation. We constructed a DynNom-based dynamic nomogram to visually display the final model. R software version 4.0.3 was used for all statistical analyses. *P* < 0.05 was considered statistically significant.

## Results

### Baseline Characteristics

After excluding the patients who did not meet the inclusion criteria, a total of 919 patients were included in this study. Among them, 804 were from the training set and 115 were from the verification set. We divided these patients into two groups based on their death or survival in 30-day. Table 1 summarizes the basic distribution of all the target patients' baseline characteristics, vital signs, comorbidities, and other indicators. In the training set, the difference in age, SBP, MBP, DBP, respiratory rate, temperature, SpO_2_, WBC count, RDW, INR, PT, anion gap, bicarbonate, blood lactic acid, serum creatinine, serum urea nitrogen, serum potassium, tumor, SAPSII score, and length of stay in the hospital between the two groups was statistically significant. In the validation set, the difference in temperature, PT, bicarbonate, blood lactic acid, congestive heart failure, pneumonia, SAPSII score, and length of stay in hospital between the two groups was statistically significant.

**Table 1:** Baseline characteristics of the study population of 30-day all-cause death.

	**Training set**			**Validation set**		
	**Survival in 30-day**	**Death in 30-day**	***P***	**Survival in 30-day**	**Death in 30-day**	***P***
**Number of patients**	500	304		53	62	
**Pathogeny**			/			0.392
Acute coronary syndrome, *n* (%)	/	/		47 (88.68)	56 (90.32)	
Valvopathy, *n* (%)	/	/		2 (3.77)	1 (1.61)	
Cardiomyopathy, *n* (%)	/	/		2 (3.77)	4 (6.45)	
Heart failure, *n* (%)	/	/		0 (0.00)	1 (1.61)	
Atrial fibrillation, *n* (%)	/	/		2 (3.77)	0 (0.00)	
**Clinical parameters**						
Age, years	77.50 ± 7.39	80.01 ± 7.51	<0.001	76.28 ± 6.47	78.56 ± 7.62	0.089
Sex, *n* (%)			0.623			0.498
Female	223 (44.60)	141 (46.38)		24 (45.28)	32 (51.61)	
Male	277 (55.40)	163 (53.62)		29 (54.72)	30 (48.39)	
Ethnicity, *n* (%)			0.279			/
White	346 (69.20)	217 (71.38)		/	/	
Black	31 (6.20)	11 (3.62)		/	/	
Others	123 (24.60)	76 (25.00)		53 (100)	62 (100)	
**Vital signs**						
Heart rate, beats/minute	87.09 ± 16.08	89.44 ± 17.29	0.051	99.75 ± 23.97	104.05 ± 19.98	0.299
SBP, mmHg	104.97 ± 13.53	100.44 ± 13.57	<0.001	124.58 ± 33.65	114.49 ± 28.03	0.083
MBP, mmHg	72.45 ± 8.99	69.70 ± 9.73	<0.001	89.36 ± 24.28	83.15 ± 21.53	0.150
DBP, mmHg	55.25 ± 8.85	53.21 ± 9.33	0.002	71.75 ± 21.96	67.48 ± 20.81	0.288
Respiratory rate, times/minute	19.50 ± 3.84	20.49 ± 4.27	<0.001	20.51 ± 6.11	23.02 ± 7.94	0.065
Temperature, °C	36.73 ± 0.76	36.53 ± 1.08	0.003	36.78 ± 0.88	36.40 ± 0.97	0.034
SpO_2_, %	96.69 ± 4.09	95.31 ± 6.78	<0.001	95.36 ± 8.62	94.48 ± 7.78	0.575
**Laboratory parameters**						
WBC count, 10^9^/L	12.50 ± 5.69	14.20 ± 7.40	<0.001	15.03 ± 5.52	15.80 ± 5.63	0.466
RDW, %	15.07 ± 1.93	15.62 ± 2.57	<0.001	14.38 ± 1.72	14.03 ± 1.54	0.262
Hemoglobin, g/dl	11.07 ± 2.18	11.08 ± 2.02	0.944	11.71 ± 2.81	11.65 ± 2.14	0.904
Hematocrit, %	33.40 ± 6.37	33.58 ± 6.04	0.704	0.36 ± 0.08	0.36 ± 0.07	0.913
Platelet, 10^9^/L	229.84 ± 110.60	223.86 ± 113.18	0.461	242.68 ± 93.47	210.02 ± 101.88	0.081
APTT, s	53.02 ± 35.10	58.47 ± 49.84	0.069	99.00 ± 65.95	94.00 ± 61.63	0.681
INR	1.78 ± 1.54	2.16 ± 2.20	0.004	1.54 ± 1.59	1.75 ± 1.03	0.403
PT, s	17.49 ± 9.65	20.11 ± 15.97	0.004	16.09 ± 3.00	19.31 ± 7.89	0.006
Anion gap, mmol/L	16.44 ± 4.47	18.84 ± 5.34	<0.001	15.99 ± 5.96	19.41 ± 6.41	0.070
Bicarbonate, mmol/L	22.11 ± 4.69	20.40 ± 6.08	<0.001	18.96 ± 5.20	15.67 ± 6.09	0.003
Glucose, mg/dl	181.99 ± 94.06	183.43 ± 106.79	0.842	181.27 ± 91.40	205.57 ± 71.03	0.212
Blood lactic acid, mmol/L	3.09 ± 2.68	4.27 ± 3.64	<0.001	4.34 ± 4.09	7.64 ± 5.73	<0.001
Serum creatinine, mg/dl	1.76 ± 1.39	2.14 ± 1.52	<0.001	1.67 ± 1.35	1.86 ± 1.20	0.420
Serum urea nitrogen, mg/dl	35.57 ± 21.61	43.71 ± 27.42	<0.001	31.51 ± 20.86	36.33 ± 23.95	0.262
Serum sodium, mg/dl	137.11 ± 4.81	136.97 ± 5.82	0.705	138.06 ± 7.44	138.96 ± 5.76	0.468
Serum potassium, mg/dl	4.32 ± 0.79	4.46 ± 1.05	0.039	6.09 ± 13.73	4.34 ± 0.78	0.319
**Comorbidities**						
Congestive heart failure, *n* (%)	109 (21.80)	70 (23.03)	0.685	3 (5.66)	14 (22.58)	0.011
Atrial fibrillation, *n* (%)	258 (51.60)	146 (48.03)	0.326	9 (16.98)	18 (29.03)	0.129
Coronary heart disease, *n* (%)	151 (30.20)	84 (27.63)	0.437	44 (83.02)	53 (85.48)	0.717
Renal failure, *n* (%)	118 (23.60)	77 (25.33)	0.579	19 (35.85)	27 (43.55)	0.401
Liver disease, *n* (%)	7 (1.40)	7 (2.30)	0.343	8 (15.09)	10 (16.13)	0.879
Stroke, *n* (%)	14 (2.80)	4 (1.32)	0.168	17 (32.08)	15 (24.19)	0.347
Tumor, *n* (%)	41 (8.20)	42 (13.82)	0.011	2 (3.77)	2 (3.23)	0.873
COPD, *n* (%)	6 (1.20)	4 (1.32)	0.886	2 (3.77)	1 (1.61)	0.469
ARDS, *n* (%)	6 (1.20)	7 (2.30)	0.229	1 (1.89)	1 (1.61)	0.911
Pneumonia, *n* (%)	156 (31.20)	98 (32.24)	0.759	40 (75.47)	27 (43.55)	<0.001
**Ejection fraction, %**	/	/	/	41.74 ± 10.89	42.04 ± 13.93	0.905
**Vasopressor**, ***n*** **(%)**	416 (83.20)	266 (87.50)	0.099	50 (94.34)	58 (93.55)	0.860
**Severity of illness**						
SAPSII score	47.36 ± 13.29	55.61 ± 14.98	<0.001	42.13 ± 8.00	59.94 ± 15.07	<0.001
**Length of stay in hospital, day**	14.72 ± 12.54	6.89 ± 6.64	<0.001	21.12 ± 13.23	6.73 ± 11.01	<0.001

*The data of the training set came from MIMIC-III database version 1.4; the data of validation set came from the patient data of ICU at the Second Affiliated Hospital and Yuying Children's Hospital of Wenzhou Medical University*.

*SBP, systolic blood pressure; DBP, diastolic blood pressure; MBP, mean blood pressure; SpO_2_, arterial oxygen saturation; WBC, white blood cell; RDW, red cell distribution width; APTT, activated partial thromboplastin time; INR, international normalized ratio; PT, prothrombin time; COPD, chronic obstructive pulmonary disease; ARDS, acute respiratory distress syndrome; SAPSII, simplified acute physiology score II*.

### Model Development

Based on the training set, we developed three models. In the traditional cox regression model, we first used the monofactor analysis to pick out 8 predictors (*P* < 0.05), the results are presented in Supplementary Table 1. Then, we screened the predictors twice using multiple regression analysis (Supplementary Table 2). The final Cox regression model was shown in Table 2, a total of 6 predictors including age, heart rate, temperature, WBC count, anion gap, and blood lactic acid. In the LASSO regression model, we determined the cutoff value based on the Loess smoothing function and the Youden index. In this way, 36 features were reduced to 4 potential predictors (Supplementary Table 3), including age, SBP, anion gap, and blood lactic acid (Table 3). According to the CoxBoost machine learning analysis of the importance of the predictors (Supplementary Table 4), we selected 17 predictors (*P* < 0.05) to construct the CoxBoost model, considering the existence of multicollinearity, we removed MBP. The final model was shown in Table 4, including age, heart rate, SBP, DBP, respiratory rate, temperature, SpO_2_, WBC count, RDW, INR, PT, anion gap, bicarbonate, blood lactic acid, serum urea nitrogen, and tumor. VIF proved there was no significant multicollinearity in all three models (VIF ≤ 3).

**Table 2:** Cox regression model.

	**HR (95%CI)**	***P***
**Clinical parameters**		
Age	1.0386 (1.0229–1.0545)	<0.0001
**Vital signs**		
Heart rate	1.0118 (1.0047–1.0189)	0.0010
Temperature	0.7894 (0.6897–0.9036)	0.0006
**Laboratory parameters**		
WBC count	1.0281 (1.0110–1.0454)	
Anion gap	1.0580 (1.0332–1.0833)	0.0012
Blood lactic acid	1.0548 (1.0168–1.0943)	<0.0001

*WBC, white blood cell*.

**Table 3:** LASSO regression model.

	**HR (95%CI)**	***P***
**Clinical parameters**		
Age	1.0386 (1.0231–1.0544)	<0.0001
**Vital signs**		
SBP	0.9846 (0.9765–0.9928)	0.0002
**Laboratory parameters**		
Anion gap	1.0588 (1.0342–1.0840)	<0.0001
Blood lactic acid	1.0433 (1.0050–1.0830)	0.0262

*SBP, systolic blood pressure*.

**Table 4:** CoxBoost model.

**Variables**	***P***
**Clinical parameters**	
Age	<0.0001
**Vital signs**	
Heart rate	0.0224
SBP	<0.0001
DBP	0.0204
Respiratory rate	0.0020
Temperature	0.0020
SpO_2_	<0.0001
**Laboratory parameters**	
WBC count	<0.0001
RDW	0.0020
INR	0.0143
PT	0.0082
Anion gap	<0.0001
Bicarbonate	0.0061
Blood lactic acid	<0.0001
Serum urea nitrogen	0.0020
**Comorbidities**	
Tumor	0.0143

*SBP, systolic blood pressure; DBP, diastolic blood pressure; SpO_2_, arterial oxygen saturation; WBC, white blood cell; RDW, red cell distribution width; INR, international normalized ratio; PT, prothrombin time*.

**Figure 2:**
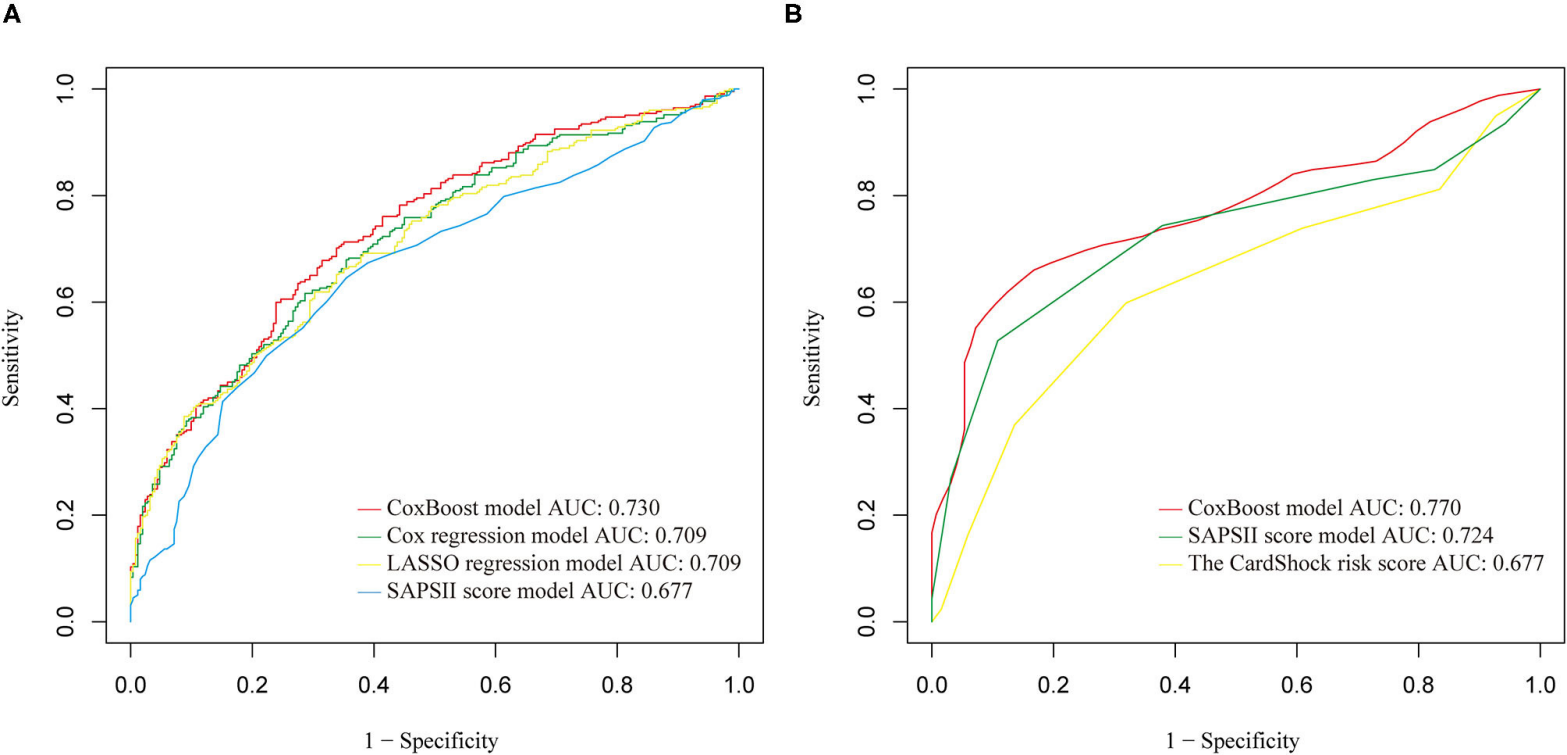
**(A)** Receiver operating characteristic (ROC) comparison of training set models. **(B)** ROC comparison of validation set models.

These three models were evaluated together with the classic SAPSII score model to choose the best one. The CoxBoost model had good discrimination (AUC: 0.730) in the training set, which was better than others (Figure 2A). Besides, using the CoxBoost model as a reference, the Cox regression model, LASSO regression model, and SAPSII score model did worse in the C index (cox regression model: 0.6835; LASSO regression model: 0.6786; SAPSII: 0.6490), NRI (cox regression model: −0.0590; LASSO regression model: −0.1480; SAPSII: −0.1630), IDI (cox regression model: −0.0040; LASSO regression model: −0.0300; SAPSII: −0.0530), and median improvement in risk score (cox regression model: −0.0040; LASSO regression model:−0.0220; SAPSII: −0.0570) (Table 5). Therefore, we choose the CoxBoost model as the target model.

**Table 5:** Model comparison.

**Model**	**C index (95%CI)**	**NRI**	**IDI**	**Median improvement in risk score**
**Training set**				
CoxBoost model	0.6958 (0.6657, 0.7259)			
Cox regression model	0.6835 (0.6529, 0.7141)*	−0.0590	−0.0040*	−0.0040
LASSO regression model	0.6786 (0.6489, 0.7084)*	−0.1480**	−0.0300**	−0.0220**
SAPSII score model	0.6490 (0.6181, 0.6799)**	−0.1630**	−0.0530**	−0.0570**
**Validation set**				
CoxBoost model	0.7713 (0.6751, 0.8675)			
SAPSII score model	0.7341 (0.6448, 0.8234)*	−0.3620*	−0.1740	−0.2380
The CardShock risk Score model	0.6628 (0.5518, 0.7738)**	−0.3480*	−0.2490*	−0.1910*

* *Compared with the CoxBoost model P < 0.05*;

** *Compared with the CoxBoost model P < 0.0001*.

*NRI, net reclassification improvement; IDI, integrated discrimination improvement*.

### Model Comparisons

In the validation set, we further evaluated and validated the CoxBoost model. The CoxBoost model was well-discriminated in the external validation set (AUC: 0.770), which was greater than the SAPSII score model (AUC: 0.724) and the CardShock risk score (Supplementary Table 5) (AUC: 0.677) (Figure 2B). In the C index, the CoxBoost model has the best performance (C index: CoxBoost model 0.7713, SAPSII score model 0.7341, CardShock risk score 0.6628, Table 5). Using the CoxBoost model as a reference, the NRI of the SAPSII score model and the CardShock risk score were −0.3620 and −0.3480, the IDI of the two models were −0.1740 and −0.2490, and the median improvement in risk scores were −0.2380 and −0.1910 respectively (Table 5), all of which suggested that the CoxBoost model was better.

### Model Presentation

We constructed a DynNom-based dynamic nomogram to visually display the final model (Table 4) (https://CoxBoost-model.shinyapps.io/DynNomapp/), which could directly obtain the patient's in-hospital mortality by inputting the values of the relevant predictors. The final model formula and its regression coefficients was as follows: survival possibility in 30-day = 0.0339 ^*^ age + 0.0104 ^*^ heart rate – 0.0070 ^*^ SBP – 0.0127 ^*^ DBP + 0.0126 ^*^ respiratory rate – 0.1959 ^*^ temperature – 0.0112 ^*^ SpO_2_ + 0.0250 ^*^ WBC count + 0.0422 ^*^ RDW + 0.0176 ^*^ INR + 0.0021 ^*^ PT + 0.0355 ^*^ anion gap – 0.0055 ^*^ bicarbonate + 0.0463 ^*^ blood lactic acid + 0.0026 ^*^ serum urea nitrogen + 0.2125 ^*^ (tumor = 1).

## Discussion

Based on the data of the training set, we developed three models altogether: the Cox regression model, the LASSO regression model, and the CoxBoost model. After extensive evaluation, the CoxBoost model was chosen as the best model. The model includes 16 predictors, which can simply and effectively predict the 30-day mortality of CS patients. In external validation, the discrimination of the CoxBoost model was better than the SAPSII and the CardShock risk score. And we developed a dynamic nomogram.

Over the years, various scoring systems have been widely used in the ICU (22, 23). However, in order to be suitable for various types of critical patients, the sensitivity and specificity of SAPSII are low. And the accuracy of the results of the assessment system depends on the experience of practitioners. There are also some small studies that used SASPAII to predict the prognosis of patients with CS (24, 25). But the integrated ICU severity score cannot accurately and reliably predict the mortality of elderly patients with CS. The CoxBoost model constructed in this study was targeted at elderly patients with CS, with higher sensitivity and specificity. In the results of our study, it can be found that the performance of the CoxBoost model was relatively better than SAPSII in the both training set and validation set.

In recent years, models have been developed specifically to assess the prognosis of patients with CS, but also have some drawbacks (26, 27). For example, the IABP-SHOCK II study established a scoring system for predicting 30-day mortality in patients with CS (9), but it is more suitable for patients with CS after emergency percutaneous coronary intervention (PCI). The CardShock risk score (10) need complicated calculation and scoring when it is used and partly depend on the subjective judgment of clinicians. Hongisto et al. evaluated the prognostic ability of two models in elderly patients with CS and the results showed that the predictive power was not very good (28). The CoxBoost model was applicable to all types of elderly patients, and external verification showed that its prediction ability was better than the CardShock risk score. Last but not least, the CoxBoost model could evaluate the short-term prognosis of elderly CS patients by simply collecting patients' vital signs, simple laboratory data, and relevant medical history at admission, all the selected predictors are objective data. The dynamic nomogram further simplified the use of the model through an easy-to-use web page.

The 16 factors that made up the CoxBoost model included all the predictive factors in the cox model and LASSO model, which showed that the CoxBoost approach has better performance and accuracy. In the factors that made up the CoxBoost model, blood lactic acid, WBC count, and anion gap were the three most important laboratory indicators. Among them, blood lactate was recognized as an important independent prognostic factor for CS (29–31). WBC count could assess the prognosis of patients, which may be related to the systemic inflammatory response caused by hypoperfusion during CS (32, 33). But the mechanism for the anion gap's prediction was unclear. Zhang et al. found that the anion gap was identified as a significant predictor of poor prognosis in patients with CS (34). It could be linked to lactic acid levels and keto, because elevated serum anion gap is usually associated with excess production of organic acid anions and reduced anion excretion (35). More experiments are needed to confirm this.

This research was a multi-center retrospective study, the main advantage was that the CoxBoost model was used for the first time to predict the 30-day mortality of elderly patients with CS. Multiple validation methods were used to compare the CoxBoost model with traditional regression analysis, LASSO regression analysis, and clinical scoring systems. The dynamic nomogram was developed to facilitate users. However, there are some limitations in this study: first, due to the relatively low incidence of CS, the sample size of patients selected in our study was relatively small. Second, there are potential differences between the training set and the validation set in terms of patient demography and research years. Third, the objective limitations of the database may produce bias. Such as the use of diagnostic codes to select patients may occur selective bias to a certain extent. Or cause a lack of potential predictors. Fourth, there was no further exploration of the database, which may make the model imperfect. We will expand the sample size and refine the sample data to conduct more in-depth research to validate our model. Overall, we believe that the model developed in this study may be useful in evaluating the prognosis of elderly patients with CS.

## Conclusion

In conclusion, this study showed that in predicting the 30-day mortality of elderly patients with CS, the CoxBoost model was superior to the Cox regression model, LASSO regression model, SAPSII, and the CardShock risk score. It is a simple and objective score that can be applied in clinical practice.

## Data Availability Statement

The raw data supporting the conclusions of this article will be made available by the authors, without undue reservation.

## Ethics Statement

The studies involving human participants were reviewed and approved by the Medical Ethics Committee of the Second Affiliated Hospital and Yuying Children's Hospital of Wenzhou Medical University. Written informed consent for participation was not required for this study in accordance with the national legislation and the institutional requirements.

## Author Contributions

KJ took responsibility for the content of the manuscript, including the data and analysis. FR had full access to all of the data in the study and take responsibility for the integrity of the data and the accuracy of the data analysis. All authors contributed substantially to the study design, data analysis and interpretation, and the writing of the manuscript. All authors contributed to the article and approved the submitted version.

## Conflict of Interest

The authors declare that the research was conducted in the absence of any commercial or financial relationships that could be construed as a potential conflict of interest.

## Publisher's Note

All claims expressed in this article are solely those of the authors and do not necessarily represent those of their affiliated organizations, or those of the publisher, the editors and the reviewers. Any product that may be evaluated in this article, or claim that may be made by its manufacturer, is not guaranteed or endorsed by the publisher.
