# Supplementary material for: Machine Learning for Prediction of Outcomes in Cardiogenic Shock
Source: Front Cardiovasc Med. 2022 May 6;9:849688. doi: 10.3389/fcvm.2022.849688 (PMC9120613; doi:10.3389/fcvm.2022.849688)
Supplement: Supplementary file 1 [file Table_1.DOCX]

**Supplement 1** Monofactor analysis of Cox regression model.

| Variables | HR (95%CI) | *P* |
| --- | --- | --- |
| **Clinical parameters** |  |  |
| Age | 1.0356 (1.0189-1.0525) | <0.0001 |
| Sex | 0.8930 (0.6898-1.1560) | 0.3902 |
| Ethnicity | 0.9631 (0.7265-1.2767) | 0.7937 |
| **Vital signs** |  |  |
| Heart rate | 1.0098 (1.0019-1.0178) | 0.0152 |
| SBP | 0.9855 (0.9716-0.9997) | 0.0448 |
| MBP | 1.0225 (0.9928-1.0531) | 0.1395 |
| DBP | 0.9753 (0.9522-0.9990) | 0.0409 |
| Respiratory rate | 1.0189 (0.9872-1.0516) | 0.2450 |
| Temperature | 0.8141 (0.7031-0.9425) | 0.0059 |
| SpO_2_ | 0.9905 (0.9694-1.0122) | 0.3885 |
| **Laboratory parameters** |  |  |
| WBC count | 1.0257 (1.0068-1.0450) | 0.0074 |
| RDW | 1.0676 (0.9983-1.1416) | 0.0562 |
| Hemoglobin | 1.0361 (0.8357-1.2847) | 0.7462 |
| Hematocrit | 0.9944 (0.9266-1.0671) | 0.8755 |
| Platelet | 0.9993 (0.9981-1.0005) | 0.2739 |
| APTT | 0.9999 (0.9968-1.0031) | 0.9745 |
| INR | 1.0065 (0.9185-1.1030) | 0.8892 |
| PT | 1.0048 (0.9917-1.0180) | 0.4755 |
| Anion gap | 1.0491 (1.0131-1.0864) | 0.0072 |
| Bicarbonate | 1.0022 (0.9727-1.0326) | 0.8855 |
| Glucose | 0.9996 (0.9983-1.0009) | 0.5257 |
| Blood lactic acid | 1.0470 (1.0047-1.0910) | 0.0290 |
| Serum creatinine | 0.9744 (0.8771-1.0824) | 0.6286 |
| Serum urea nitrogen | 1.0047 (0.9983-1.0111) | 0.1495 |
| Serum sodium | 1.0017 (0.9779-1.0261) | 0.8905 |
| Serum potassium | 0.9919 (0.8553-1.1504) | 0.9148 |
| **Comorbidities** |  |  |
| Congestive heart failure | 1.0315 (0.7678-1.3859) | 0.8366 |
| Atrial fibrillation | 0.8377 (0.6689-1.0491) | 0.1230 |
| Coronary heart disease | 1.0503 (0.7819-1.4107) | 0.7447 |
| Renal failure | 0.8645 (0.6268-1.1924) | 0.3748 |
| Liver disease | 1.6282 (0.7484-3.5421) | 0.2190 |
| Stroke | 0.5815 (0.2141-1.5793) | 0.2875 |
| Tumor | 1.3040 (0.9109-1.8667) | 0.1470 |
| COPD | 0.8163 (0.2913-2.2872) | 0.6994 |
| ARDS | 1.9517 (0.8921-4.2697) | 0.0941 |
| Pneumonia | 1.0089 (0.7748-1.3137) | 0.9475 |

**Abbreviations:** SBP: systolic blood pressure; DBP: diastolic blood pressure; MBP: mean blood pressure; SpO_2_: arterial oxygen saturation; WBC: white blood cell; RDW: red cell distribution width; APTT: activated partial thromboplastin time; INR: international normalized ratio; PT: prothrombin time; COPD: chronic obstructive pulmonary disease; ARDS: acute respiratory distress syndrome.
