# Supplementary material for: Machine Learning for Prediction of Outcomes in Cardiogenic Shock
Source: Front Cardiovasc Med. 2022 May 6;9:849688. doi: 10.3389/fcvm.2022.849688 (PMC9120613; doi:10.3389/fcvm.2022.849688)
Supplement: Supplementary file 2 [file Table_2.DOCX]

**Supplement 2** Multiple regression analysis of Cox regression model.

| Variables | HR (95%CI) | *P* |
| --- | --- | --- |
| **Clinical parameters** |  |  |
| Age | 1.0361 (1.0200-1.0524) | <0.0001 |
| **Vital signs** |  |  |
| Heart rate | 1.0117 (1.0043-1.0193) | 0.0021 |
| SBP | 0.9908 (0.9815-1.0002) | 0.0554 |
| DBP | 0.9869 (0.9718-1.0023) | 0.0954 |
| Temperature | 0.7912 (0.6903-0.9069) | 0.0008 |
| **Laboratory parameters** |  |  |
| WBC count | 1.0276 (1.0107-1.0449) | 0.0013 |
| Anion gap | 1.0530 (1.0283-1.0782) | <0.0001 |
| Blood lactic acid | 1.0457 (1.0077-1.0852) | 0.0181 |

**Abbreviations:** SBP: systolic blood pressure; DBP: diastolic blood pressure; WBC: white blood cell.
