# Supplementary material for: Machine Learning for Prediction of Outcomes in Cardiogenic Shock
Source: Front Cardiovasc Med. 2022 May 6;9:849688. doi: 10.3389/fcvm.2022.849688 (PMC9120613; doi:10.3389/fcvm.2022.849688)

**Supplement 3** Predictors selection of LASSO regression model**.**

| Variables | Coefficients |
| --- | --- |
| **Clinical parameters** |  |
| Age | 0.0101879293847151 |
| **Vital signs** |  |
| SBP | -0.00395642469182494 |
| **Laboratory parameters** |  |
| Anion gap | 0.0392879972378275 |
| Blood lactic acid | 0.0163715547957477 |

**Abbreviations:** SBP: systolic blood pressure.


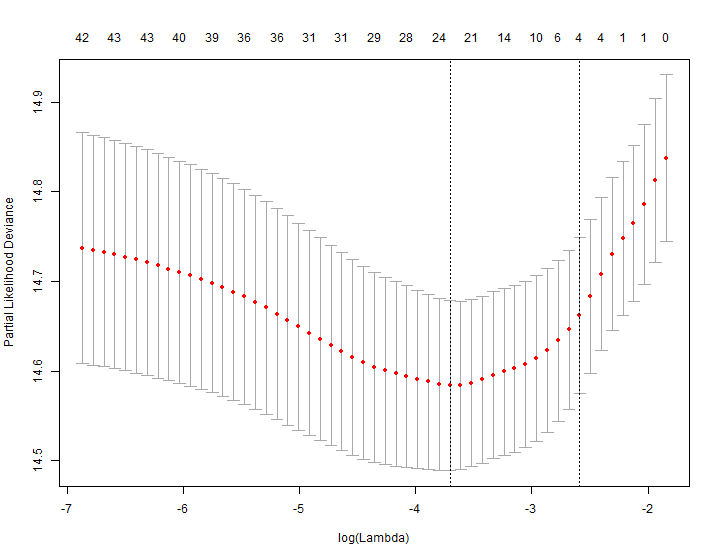

Supplement: Supplementary file 3 [file Table_3.DOCX]
