# Supplementary material for: Machine Learning for Prediction of Outcomes in Cardiogenic Shock
Source: Front Cardiovasc Med. 2022 May 6;9:849688. doi: 10.3389/fcvm.2022.849688 (PMC9120613; doi:10.3389/fcvm.2022.849688)
Supplement: Supplementary file 4 [file Table_4.DOCX]

**Supplement 4** Predictors selection of CoxBoost model.

| Variables | *P* |
| --- | --- |
| **Clinical parameters** |  |
| Age | <0.0001 |
| Sex | 0.4082 |
| Ethnicity | 0.5347 |
| **Vital signs** |  |
| Heart rate | 0.0224 |
| SBP | <0.0001 |
| MBP | 0.0020 |
| DBP | 0.0204 |
| Respiratory rate | 0.0020 |
| Temperature | 0.0020 |
| SpO_2_ | <0.0001 |
| **Laboratory parameters** |  |
| WBC count | <0.0001 |
| RDW | 0.0020 |
| Hemoglobin | 0.8449 |
| Hematocrit | 0.8265 |
| Platelet | 0.2367 |
| APTT | 0.3265 |
| INR | 0.0143 |
| PT | 0.0082 |
| Anion gap | <0.0001 |
| Bicarbonate | 0.0061 |
| Glucose | 0.5755 |
| Blood lactic acid | <0.0001 |
| Serum creatinine | 0.2755 |
| Serum urea nitrogen | 0.0020 |
| Serum sodium | 0.9449 |
| Serum potassium | 0.6673 |
| **Comorbidities** |  |
| Congestive heart failure | 0.9592 |
| Atrial fibrillation | 0.0590 |
| Coronary heart disease | 0.5959 |
| Renal failure | 0.4327 |
| Liver disease | 0.2633 |
| Stroke | 0.1000 |
| Tumor | 0.0143 |
| COPD | 0.9796 |
| ARDS | 0.0816 |
| Pneumonia | 0.8878 |

**Abbreviations:** SBP: systolic blood pressure; DBP: diastolic blood pressure; MBP: mean blood pressure; SpO_2_: arterial oxygen saturation; WBC: white blood cell; RDW: red cell distribution width; APTT: activated partial thromboplastin time; INR: international normalized ratio; PT: prothrombin time; COPD: chronic obstructive pulmonary disease; ARDS: acute respiratory distress syndrome.
