# Supplementary material for: Machine Learning for Prediction of Outcomes in Cardiogenic Shock
Source: Front Cardiovasc Med. 2022 May 6;9:849688. doi: 10.3389/fcvm.2022.849688 (PMC9120613; doi:10.3389/fcvm.2022.849688)
Supplement: Supplementary file 5 [file Table_5.DOCX]

**Supplement 5** The CardShock risk Score.

| Variable | CardShock risk Score |
| --- | --- |
| Age >75 years | 1 |
| Confusion at presentation | 1 |
| Previous MI or CABG | 1 |
| ACS aetiology | 1 |
| LVEF <40% | 1 |
| Blood lactate |  |
| <2 mmol/L | 0 |
| 2-4 mmol/L | 1 |
| >4 mmol/L | 2 |
| eGFR _CKD.EPI_ |  |
| >60 mL/min/1.73 m2 | 0 |
| 30-60 mL/min/1.73 m2 | 1 |
| <30 mL/min/1.73 m2 | 2 |
| Maximum points | 9 |

**Abbreviations:** MI: myocardial infarction; CABG: coronary artery bypass grafting; ACS: acute coronary syndrome; eGFR _CKD.EPI_: estimated glomerular filtration rate by the Chronic Kidney Disease Epidemiology Collaboration formula.
